# HybGrip: a synergistic hybrid gripper for enhanced robotic surgical instrument grasping

**DOI:** 10.1007/s11548-024-03245-5

**Published:** 2024-08-21

**Authors:** Jorge Badilla-Solórzano, Sontje Ihler, Thomas Seel

**Affiliations:** Institute of Mechatronic Systems, Leibniz University Hannover, Hannover, Germany

**Keywords:** Hybrid gripper, Surgical instrument grasping, Granular jamming gripper, Robot-assisted surgery, Robotic scrub nurse

## Abstract

**Purpose:**

A fundamental task of a robotic scrub nurse is handling surgical instruments. Thus, a gripper capable of consistently grasping a wide variety of tools is essential. We introduce a novel gripper that combines granular jamming and pinching technologies to achieve a synergistic improvement in surgical instrument grasping.

**Methods:**

A reliable hybrid gripper is constructed by integrating a pinching mechanism and a standard granular jamming gripper, achieving enhanced granular interlocking. For our experiments, our prototype is affixed to the end-effector of a collaborative robot. A novel grasping strategy is proposed and utilized to evaluate the robustness and performance of our prototype on 18 different surgical tools with diverse geometries.

**Results:**

It is demonstrated that the integration of the pinching mechanism significantly enhances grasping performance compared with standard granular jamming grippers, with a success rate above 98%. It is shown that with the combined use of our gripper with an underlying grid, i.e., a complementary device placed beneath the instruments, robustness and performance are further enhanced.

**Conclusion:**

Our prototype’s performance in surgical instrument grasping stands on par with, if not surpasses, that of comparable contemporary studies, ensuring its competitiveness. Our gripper proves to be robust, cost-effective, and simple, requiring no instrument-specific grasping strategies. Future research will focus on addressing the sterilizability of our prototype and assessing the viability of the introduced grid for intra-operative use.

## Introduction

The world faces an ever-growing scarcity of healthcare workers [1, 2]. The development and study of autonomous surgery assistants, i.e., robotic scrub nurses (RSN), is desirable to alleviate staff shortages in medical centers [3, 4]. RSN must fulfill a variety of tasks, such as surgical instrument detection and grasping. Traditionally, efforts to develop a functional RSN rely mainly on magnetic grippers [5–8], while commercial mechanical grippers are employed to a lesser extent [9, 10]. However, these technologies have limitations. Magnetic grippers cannot directly manipulate nonmagnetic tools and may introduce undesirable effects like strong force fluctuations and residual magnetism, leading to unintuitive interactions. On the other hand, mechanical grippers tend to be costly, lack adaptability to various geometries, and require precise positioning. These drawbacks, together with the diversity of materials and shapes found in typical surgery sets, pose a significant challenge. Thus, robotic grasping for surgical instruments remains an active research topic.

Among modern solutions found in the literature, pinching mechanisms, i.e., grippers based on clamping motions, are particularly relevant. Evidence of this is the work of Wagner et al. [11], who introduce an end-effector for handling tools for minimally invasive surgery. Other studies focus on grasping open-surgery instruments. Heibeyn et al. [10] design and evaluate a pinching gripper for the manipulation of 16 different open-surgery tools. The gripper specializes in handling cylindrical bodies and is effective for the employed surgery set. Kim et al. [12] propose a novel pinching mechanism and consider 12 open-surgery instruments. They achieve 67% pick-up success rate, with a mean pick-up time of 15.81 s. In general, pinching mechanisms specialize in specific geometries, e.g., cylindrical bodies, and are incompatible with the diversity of shapes present in typical surgery sets (see “Dealing with thin or flat instruments” section).

To address a wide range of geometries, the use of universal grippers becomes interesting. Traditional universal grippers typically rely on magnetism or suction. While magnetic technology is incompatible with nonmagnetic materials, suction-based systems are inadequate for handling thin objects commonly found in surgery sets. More advanced universal grippers are based on *granular jamming* and are known as granular jamming grippers (GJGs) [13]. They rely on a membrane filled with granular material, which is vacuumized to solidify the granules to perform grasping. They stand out for being simple and adaptable to multiple geometries [14, 15]. The use of this technology for open-surgery instrument grasping from a flat surface is studied by Schäfer et al. [16]. Their experiments include six different tools, and success rates of 95% on a pick-up task and 50% on a transfer task are achieved. The authors highlight the need for proper adjustment of the GJG’s parameters, e.g., granular mixture and membrane thickness, for adequate performance. In general, GJGs require the granular material to properly surround the objects to successfully grasp them, which is not always feasible depending on their geometry and the underlying surface.

**Fig. 1:**
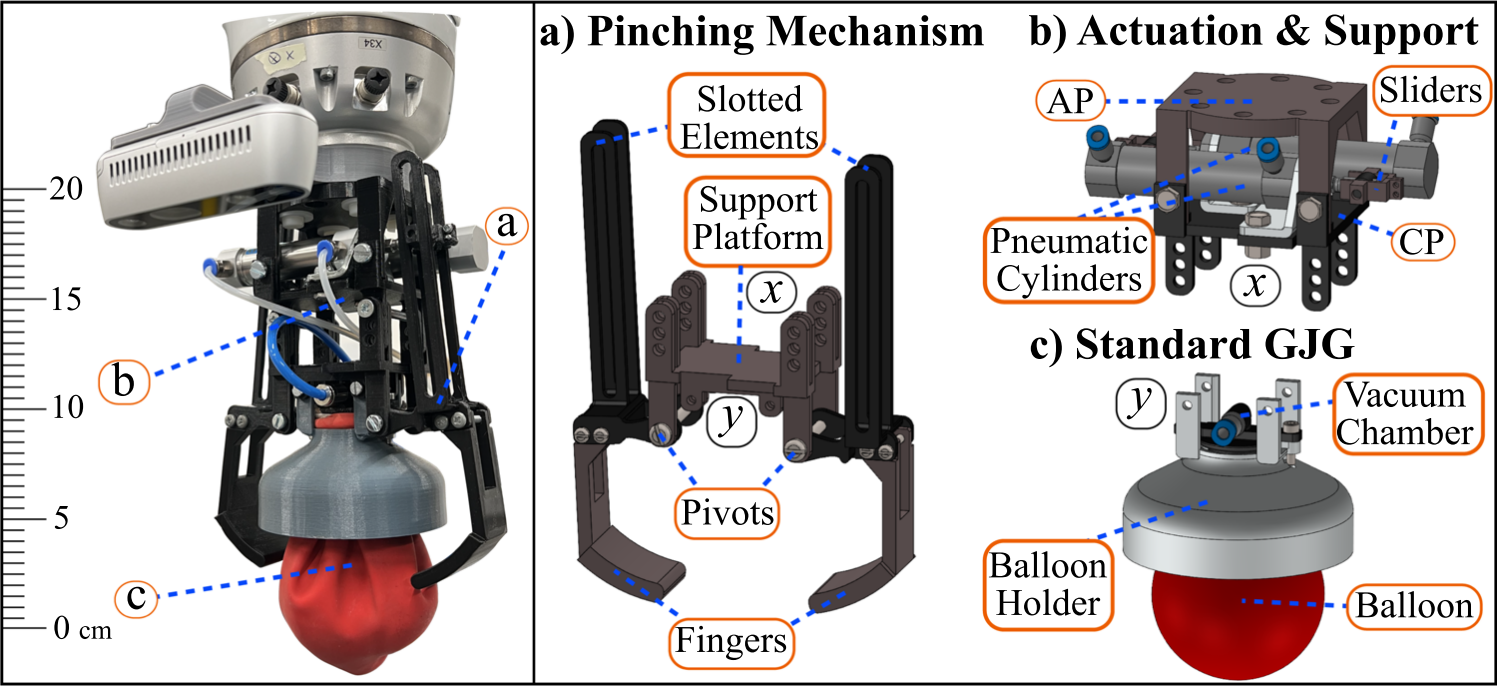
HybGrip and its components. The HybGrip (left) includes three subsystems (right): **a** pinching mechanism, **b** actuation and support subsystem, and **c** standard GJG. The sliders are confined in the slotted elements to transfer motion. AP: attachment platform, CP: central platform. x, y: coupling points

In this work, a novel instrument gripper that combines the reliability of pinching mechanisms with the adaptation capabilities of GJGs is introduced. With this combination, the grasping performance is improved by actively forcing the granules to adapt to the geometry of the instruments, achieving enhanced interlocking [14]. Our prototype is named HybGrip (see Fig. 1) and it is evaluated on a complete surgery set for wisdom teeth extraction with 18 tools (see “Dealing with thin or flat instruments” section). The high performance of our prototype suggests that it could imply a significant milestone for surgical instrument grasping and the field of universal gripping in general. The contributions of this work are: (1) the introduction of an effective hybrid gripper that improves upon standard GJGs and guarantees both robustness and reliability, (2) the proposition of an effective grasping strategy with an associated mean pick-up time of 3.7 s, (3) the thorough evaluation of our gripper in the task of surgical instrument grasping, with success rate above 98%, (4) the introduction of a flat underlying grid as a complementary tool, tailored for our gripper, that significantly enhances grasping performance, and (5) the publication of our design and other relevant information for the scientific community (https://github.com/Jorebs/HybGrip).

## HybGrip: design and challenges

This section introduces the HybGrip’s working principle, its components, and a novel grasping strategy. Challenges for GJGs in tool grasping are also explained.

### Working principle

**Fig. 2:**
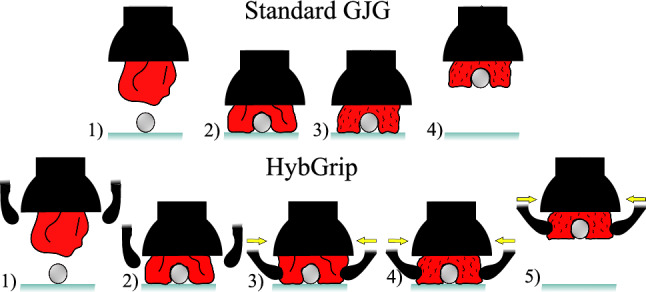
Grasping stages of a standard GJG (top) and the HybGrip (bottom). Standard GJG: (1) object is approached, (2) relaxed membrane is pressed against object, (3) gripper is vacuumized, (4) object is lifted. HybGrip: (1) object is approached, (2) relaxed membrane is pressed against object, (3) pinching mechanism is activated, (4) gripper is vacuumized, 5) object is lifted. White arrows: vertical motion. Blue arrows: vacuum. Yellow arrows: pinching force

Standard GJGs include granular material contained in a flexible membrane. With the use of a filter, vacuum can be applied to extract the air inside the membrane, generating a jamming effect that solidifies the granular material. This solidification can be applied while the membrane surrounds an object to grasp it (see Fig. 2). According to Amend et al. [14], three effects contribute to grasping using granular jamming: (1) friction between the object and the membrane, (2) suction produced by an airtight seal, and (3) interlocking between the object and the solidified granular material. With the HybGrip, these principles are exploited, especially the interlocking, by exerting lateral pinching forces on the membrane to rearrange the granules around the object (see Fig. 2).

### Construction of the HybGrip

The HybGrip (Fig. 1) is actuated using two double-acting pneumatic cylinders (stroke: 16 mm, internal diameter: 30 mm), purchased from Festo SE & Co. KG (50.5 EUR). As an elastic membrane, a commercial balloon from the company Karaloon GmbH (5.22 EUR) is employed, filled with 62.5 g of medium-fine coffee powder of the brand Dallmayr Prodomo® (6.99 EUR). A piece of cotton fabric is used as a filter in the GJG. Other components are 3D printed with PLA using an Ultimaker®. The estimated total cost is below 90 EUR. The related CAD files and further details are available at https://github.com/Jorebs/HybGrip.

### Dealing with thin or flat instruments

GJGs are limited by their capacity to embrace a target object, making certain geometries inherently more challenging to grasp. Different works [14, 15] have studied the effect of the object size on a GJG’s performance, showcasing that grasping larger objects is comparatively difficult. However, these studies do not consider thin objects (thickness < 20 mm) common in surgery sets. As presented in Table 1, our considered instruments vary significantly in dimensions and mass.

**Table 1:** Surgery set for wisdom teeth extraction

Instrument	ID	L	W	GP	T	Mass	Instrument	ID	L	W	GP	T	Mass (g)
		(mm)				(g)			(mm)				
	**00**	141	25	**67**	12.0	51.6		**09**	153	15	83	10.5	23.2
	**01**	179	$\overline{{\textbf {81}}}$	107	$\overline{{\textbf {17.9}}}$	$\overline{{\textbf {105.0}}}$		**10**	148	18	85	7.1	21.2
	**02**	178	17	91	6.7	33.7		**11**	218	29	129	6.0	62.5
	**03**	141	12	91	3.6	20.1		**12**	220	29	$\overline{{\textbf {135}}}$	6.9	61.5
	**04**	$\overline{{\textbf {222}}}$	22	106	5.0	26.1		**13**	208	73	135	5.0	62.6
	**05**	205	8.1	120	8.0	34.3		**14**	150	69	99	**3.0**	28.3
	**06**	185	13	95	5.4	23.0		**15**	142	47	91	4.2	25.0
	**07**	175	**5**	85	5.4	21.7		**16**	122	63	83	3.4	**19.3**
	**08**	159	20	109	6.2	26.8		**17**	**113**	63	78	4.4	19.7

The bold values (excluding those in the ID columns) represent the maximum and minimum values of the corresponding column. The bold values with underline represent minimum values while those with overline represent maximum values

Grasping points (GP), depicted in red, are heuristically defined and measured from the handle. Instruments 00 and 04 include nonmagnetic materials. Maximum and minimum values are highlighted. L: length. W: width. T: thickness at GP

**Fig. 3:**
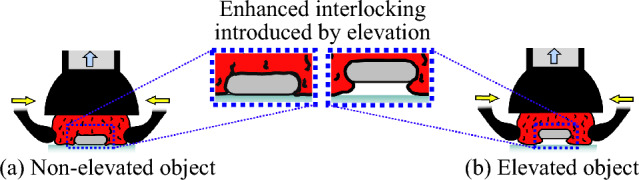
Interlocking between a thin object and the granules. **a** The HybGrip achieves a good level of interlocking when grasping from a flat surface. **b** The interlocking is further improved when the target object is elevated

**Fig. 4:**
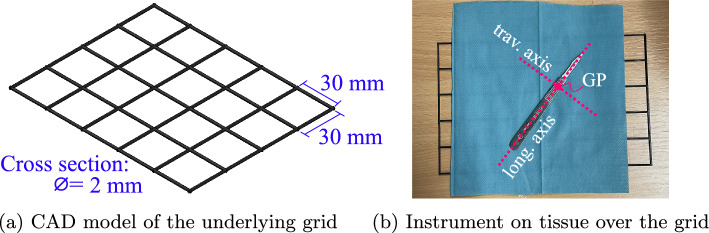
Underlying 3D-printed grid for the tools to facilitate grasping. **a** Dimensions are ${150\,\textrm{mm}} \times {120\,\textrm{mm}}$, with circular cross section. **b** The grid is placed between the table and a tissue on which the tools lay. The instruments’ longitudinal (long.) and traverse (trav.) axes, and grasping points (GP) are depicted

Preliminary evaluation of the HybGrip shows that grasping instruments under 10 mm thick from a flat surface is challenging, even with the pinching mechanism. The introduction of a small elevation of the tools from the surface proved to allow for enhanced interlocking, facilitating grasping (see Fig. 3). To optimize this, a simple solution is proposed: using a flat, thin grid under the instruments (see Fig. 4). The grid has 30 mm square apertures, which prevent tools from lodging and enable the desired elevation.

### Determination of the grasping points

The grasping point (GP) for each instrument (see Table 1, Fig. 4b) is heuristically determined, avoiding the handles and tips of the tools. This maintains the handles exposed for potential transference to a human and avoids the sharp edges of the tips to guarantee the membrane’s longevity.

### Grasping strategy

**Fig. 5:**
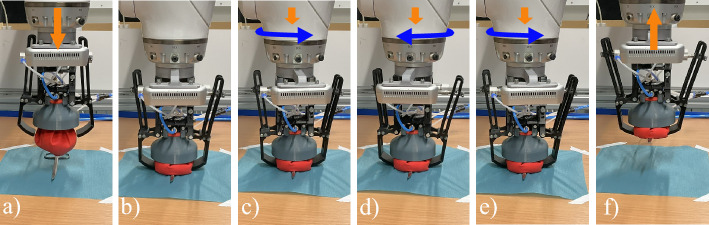
Grasping strategy using the HybGrip. **a** Initial position. **b** Gripper is lowered to 1.5 mm above $h_\textrm{p}$, pinching mechanism is activated. **c** Descent of 0.5 mm, rotation of 0.1 rad around vertical axis. **d** Descent of 0.5 mm, rotation of − 0.2 rad. **e** Descent of 0.5 mm, rotation of 0.2 rad. **f** Original orientation is restablished, vacuum is applied, and instrument is lifted. Orange arrows: translation. Blue arrows: rotation

An innovative grasping strategy composed of a reciprocating rotatory motion and a simultaneous linear motion is introduced. This promotes: (1) the proper closure of the pinching mechanism and (2) an adequate distribution of the granules around the instrument. The strategy demands finding the pinching height $h_\textrm{p}$, at which the grasping occurs, which is experimentally determined. $h_\textrm{p}$ is defined as the minimum height of the HybGrip at which no contact between fingers and the surface (or grid) occurs during the pinching. Figure 5 depicts our strategy, with an associated mean pick-up time of 3.7 s.

## Experiments and results

In this section, the evaluation of the HybGrip is described. Three cases are considered: (1) standard GJG, i.e., HybGrip without the pinching mechanism, (2) the HybGrip grasping from a flat surface (HG), and (3) the HybGrip used with our proposed grid (HGG). Unless otherwise indicated, the experiments consider an ideal grasping pose (IGP), i.e., grasping from the instrument’s GP with the pinching mechanism aligned with its longitudinal axis (see Figs. 4b, 5a).

### Holding force test

**Fig. 6:**
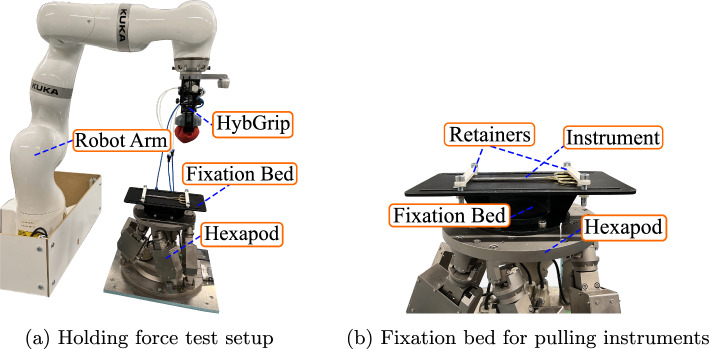
Setup used for the holding force test. **a** Robot arm equipped with HybGrip to grasp instruments and measure vertical forces. **b** Fixation bed to keep an approximately constant instrument pose during measurement and eliminate external torques. The gripper remains static during measuring phase

Holding force tests are undergone by quantifying the maximum vertical force applied to a grasped tool without making it fall. The HybGrip is attached to the end-effector of an *LBR iiwa 14 r820* (KUKA®) robot (Fig. 6a) equipped with internal force-torque sensors. This robot is used both as a manipulator and a force-measuring device. An instrument fixation bed (Fig. 6b) is designed and attached to an *H-824 6-Axis Hexapod* (Physik Instrumente®) to generate stable vertical motions. The force measurement requires three steps: (1) our grasping strategy (“Grasping strategy” section) is applied on an instrument fixed to the bed without lifting it, (2) the recording of the external vertical force on the end-effector is started, (3) the hexapod is lowered until the tool is released. Figure 6 describes our full setup.

**Fig. 7:**
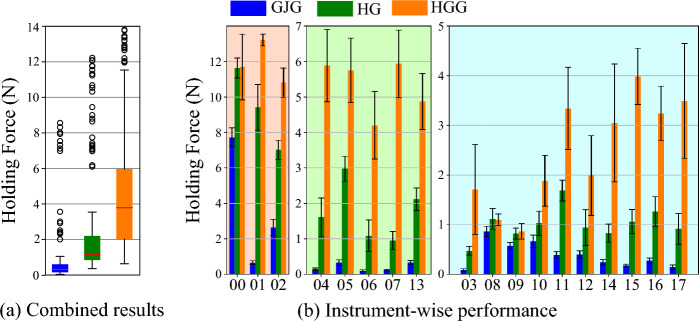
Results of the holding force test. **a** Box plots for the combined results for all instruments. **b** Mean holding forces per instrument. Three different vertical scales are used for better visualization. *X*-axes: instrument IDs. GJG: standard granular jamming gripper. HG: HybGrip. HGG: HybGrip with grid

For each instrument, eight measurements per case (GJG, HG, HGG) are performed. The results of the holding force test are presented in Fig. 7. The box plots indicate small values for the GJG, depicting its poor performance. Contrastively, a significant improvement is observed for the HybGrip, demonstrating the importance of the pinching mechanism. An even stronger improvement is observed for the HGG, indicating the positive effect of employing the proposed grid. Using the combined data for all instruments, we corroborate these conclusions by applying Student’s *t* tests for paired samples. With $p < 0.01$, statistically significant differences between the holding forces of the GJG and HG cases (test 1), and the HG and HGG cases (test 2) are determined. The instrument-specific values in Fig. 7b) are consistent with these conclusions.

### Pick-up success rate

**Fig. 8:**
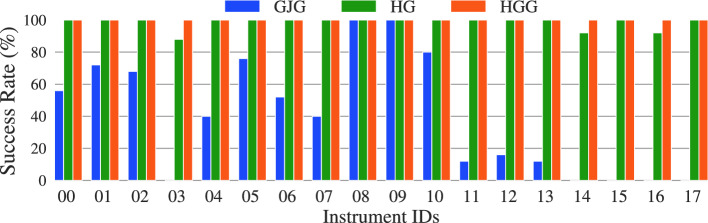
Pick-up success rate using IGP for 25 trials per instrument. Mean success rates for the standard granular jamming gripper (GJG), the HybGrip (HG), and the HybGrip with grid (HGG) are 40.2%, 98.4%, and 100%, respectively

To quantify the pick-up success rate, the instruments are placed on a surgical tissue fixed to a table (see Fig. 5) and are lifted using an IGP. After lifting each tool, the robot performs a shake, corresponding to a reciprocating motion with an amplitude of 80 mm and a maximum acceleration of ${6.8}\,{\hbox {m}/\hbox {s}^{2}}$. For each case, each instrument is grasped 25 times, and success is declared only if it remains grasped after being shaken. Figure 8 depicts the results. The data indicate a clear superiority of the HybGrip compared to the standard GJG, with success rates of 98.4% and 40.2%, respectively. The use of the underlying grid further improves the performance of the HybGrip, leading to successful grasps in all trials. While the standard GJG fails to adapt to some geometries, e.g., Instruments 03 and 14, the HybGrip is capable of grasping all the considered instruments.

### Robustness against displacements

**Fig. 9:**
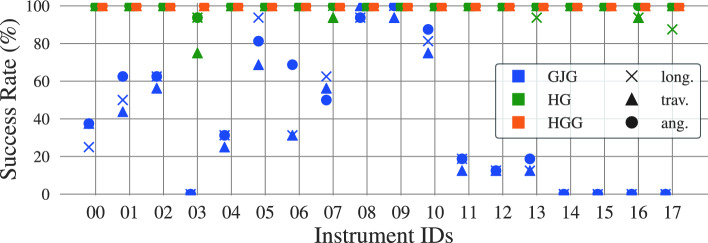
Robustness against displacements. Deviations with respect to the IGP are considered along the instruments’ longitudinal (long.) and traversal (trav.) axes ($\pm {5\,\textrm{mm}}$). Angular (ang.) displacements are also contemplated ($\pm 30^{\circ }$). The GJG and HybGrip (HG) achieve success rates of 37.5% and 97.8%, respectively. The HybGrip with grid (HGG) achieves success in all 864 trials

The grasping performance under the consideration of linear and angular deviations from the IGP is also studied. Two points located along the tool’s longitudinal axis and two along its traverse axis (see Fig. 4b) are selected. The points are located at both sides of the GP at a distance of 5 mm. By determining the pick-up success rate from these points, the robustness of our gripper against longitudinal and traversal displacements is quantified. For angular deviations, grasping is performed on the GP using $30^{\circ }$ and $-30^{\circ }$ angular displacements with respect to the IGP. For each instrument and case, eight grasping trials per point and orientation are performed for a total of 16 trials for longitudinal (long.), traverse (trav.), and angular (ang.) displacements. Success is declared if the grasped is maintained after applying the shake described in section “Pick-up success rate”. Figure 9 depicts the results, which are consistent with those previously obtained: the HybGrip greatly improves upon the standard GJG, and the use of the grid further enhances its robustness against displacements from the IGP.

## Discussion

Our experiments show a clear trend: employing the pinching mechanism enhances the holding force, grasping performance, and overall robustness, making the HybGrip superior to the standard GJG. This is attributed to the increased granular interlocking achieved with our prototype. Our GJG achieves comparable grasping capacity (above 50% success rate with eight different tools) to that of similar grippers in the literature (50% mean success rate with six tools [16]). Thus, our GJG is considered a suitable experimental baseline to highlight the benefits of the integration of a pinching mechanism into the HybGrip.

Our prototype also offers advantages over other technologies. Typical pinching grippers are negatively affected by substantial angular displacements ($\sim 30^{\circ }$). As shown in Fig. 9, our prototype remains robust against orientation deviations, thanks to the soft contact between the gripper and the grasped tool. There might exist potential cases in which larger deviations ($\sim 60^{\circ }$) occur. In such rare scenarios, the performance of the HybGrip will significantly drop, unlike that of a GJG, which tends to remain constant. Furthermore, the HybGrip is compatible with nonmagnetic materials (Instruments 00 and 04) and thin geometries (Instruments 04, 05, 06, and 07), which are incompatible with alternative technologies such as magnetic and suction grippers, respectively.

The combined use of the underlying grid with the HybGrip proved advantageous, resulting in improved grasping performance. Additional experiments showed that the grid is also beneficial when combined with the GJG. However, the associated improvement is modest compared to that of the HybGrip, even when the grid is not employed. This underscores how the proposed grid is merely a helpful accessory and not a primary solution for the grasping problem.

The results indicate that for certain tools, e.g., Instruments 04 and 16, similar mean holding forces (Fig. 7) are observed, while very different success rates (Fig. 8) are determined. This is attributed to fundamental differences in the corresponding experiments. Specifically, the presence of external torques enhanced by the application of the strong shake during the assessment of the pick-up success rate is absent during the holding force test (see Fig. 6), which can lead to low success rates even for tools with non-neglectable holding forces. Moreover, when comparing Figs. 8 and 9, surprising phenomena can be found, such as unexpected differences in success rates for the GJG when angular displacements occur. This could be attributed to the limited number of trials performed for each tool (25 and 16 in Experiments 1 and 2, respectively), as well as the asymmetric shape of the balloon (see Fig. 1) and the unpredictable granule arrangement.

Beyond surgical instrument grasping, the HybGrip’s versatility shows promise for other grasping tasks. With an estimated total cost below 90 EUR (without the vacuum source), the current prototype is affordable and available for further research for the scientific community. Despite the drawback of necessitating a rough alignment with the tool’s longitudinal axis (ang. displacement $<30^{\circ }$), its enhanced performance clearly surpasses that of the standard GJG.

Our evaluation involving 18 different instruments exceeds the scope of previous related studies by Schäfer et al. [16] (6 tools), Kim et al. [12] (12 tools), and Heibeyn et al. [10] (16 tools). Even with a larger instrument set, our prototype exhibits remarkable performance, as summarized in Table 2.

**Table 2:** Summarized grasping results

	Number of Trials	GJG	HybGrip	HybGrip+Grid
Ideal grasping pose (IGP)	450	40.2%	98.4%	100%
Robustness	864	37.5%	97.8%	100%

The values correspond to the pick-up success rates determined with an IGP and with deviations from it (robustness)

With 98.4% success rate, and under the consideration of strong shaking, the HybGrip outperforms the grippers presented by Schäfer et al. [16] (success rates of 95% and 50% for pick-up and transport tasks) and Kim et al. [12] (67% success rate). In this last work, the associated grasping time corresponded to 15.81 s, more than four times our associated pick-up time (3.7 s) with the proposed grasping strategy. These achievements are attained while conducting an extensive evaluation with a large number of trials, i.e., over 1000 grasping attempts per gripper, and employing a more extensive surgery set.

Regarding the design of Heibeyn et al. [10], while it proves to be effective and robust, achieving success in all trials, the number of employed grasping attempts is limited. This raises concerns about the reliability of the results. In contrast, our experiments imply dozens of trials per instrument (see Table 2). The success rate achieved by the HybGrip ($> 97.5\%$) is comparable with that of our peers’ prototype with a slightly larger surgery set (16 vs. 18 instruments) and more trials. With the use of the underlying grid, the grasping performance can be fully matched. Moreover, their gripper is specialized for grasping cylindrical bodies, and for the task of bin picking. Conversely, our prototype is adaptable to multiple geometries, and our strategy aims to address not only picking tasks but future robot–human interaction. It can then be said that each prototype could serve different niche applications, not necessarily competing with one another.

Despite defining the GP (“Determination of the grasping points”) in such a way that the tools’ tips are avoided, the significant grasping area of the balloon implies a certain degree of contact with the tips. This is especially critical for tools including sharp blades like Instrument 03 (see Table 1). Throughout our experiments, the employed membrane remained undamaged, proving its functionality even in the presence of sharp objects. Future work demands experiments addressing the compatibility of our prototype in the presence of pointy objects, e.g., needles, during grasping.

The observed performance justifies further investigation regarding the integrability of our prototype and the grid in an actual operating room, which escapes the scope of this work. Design modifications will be studied to address the gripper’s sterilizability, which may benefit from the higher simplicity of the HybGrip’s pinching mechanism compared to commercial grippers. The requirement for only two gripper positions, i.e., open and closed, and the absence of direct contact between the tools and the mechanism make the design easier to implement and more adaptable to sterilization demands. Furthermore, the material choice and manufacturing method for the grid is flexible, allowing for the exploration of cost-effective options compatible with surgical environments.

## Conclusions

The HybGrip, a novel hybrid gripper for surgical instrument grasping for RSN, is introduced and extensively evaluated. This prototype surpasses the standard GJG, boasting a remarkable 98.4% success rate across 18 instruments with various geometries. Its performance rivals, if not exceeds, that of comparable modern solutions, showcasing its potential. We avoid relying on instrument-specific grasping approaches by employing a single original grasping strategy with a competitive pick-up time of 3.7 s. With the introduction of an underlying grid for elevating the tools from the flat surface, the performance of our prototype is further enhanced, achieving success in all performed grasping trials.

Beyond this present work, which focuses on the development of new grasping concepts, a thorough study of our prototype’s limitations, including maximum admissible pose deviations and performance variance, will be performed in the future to allow for a more thorough assessment of its robustness and an improved comparison with other grasping technologies. Future research endeavors will also focus on integrating the HybGrip with our previous work [3, 4] to achieve vision-based tool grasping. Improvements in the sterilizability of the HybGrip will be made for its integration as part of a functional RSN to be used in an actual operating room. Finally, the parameters of the gripper will be optimized to guarantee further reliability and safety.
